# A Case of Massive Air Leakage After Lung Cancer Surgery Successfully Treated With the "Tachosuture" Technique and Intensive Management Under One-Lung Ventilation

**DOI:** 10.7759/cureus.111688

**Published:** 2026-06-28

**Authors:** Yoshitaka Fujii, Tatsuya Nishida

**Affiliations:** 1 Department of Thoracic Surgery, Ishikiriseiki Hospital, Osaka, JPN

**Keywords:** intensive management, low pulmonary function, massive air leakage, one-lung ventilation, pneumatocele, tachosuture technique

## Abstract

Massive air leakage after lung resection can be difficult to control, especially in patients with vulnerable lung tissues due to underlying diseases. TachoSil^®^ (Corza Medical, Linz, Austria) patch is applied to the site of air leakage during surgery to control air leakage; however, in cases of massive air leakage, it may peel off before the tissue has healed. This report describes a method for securely positioning the TachoSil^®^ patch at the site of air leakage to control massive air leakage after lung resection. The patient was a 50-year-old man with a history of heavy smoking and alcoholic liver disease. He underwent a thoracoscopic resection of the left lower lobe and mediastinal lymph node dissection for the adenocarcinoma of the lower lobe of the left lung, cT1cN0M0 stage IA3, which was detected during a health checkup. Postoperatively, in the intensive care unit (ICU), the patient developed massive air leakage with uncontrolled subcutaneous emphysema expansion triggered by coughing. Therefore, a second surgery was performed on the same day, revealing that a pneumatocele had developed, which was causing air leakage.

First, a TachoSil^®^ patch was applied over the pleural defect, and stitches were added over it. Next, the normal pleura at both ends of the pneumatocele was sutured to sew it inside. Finally, an additional layer of TachoSil^®^ patch was applied over it. The patient was then managed in the ICU under analgesia and sedation for three days with one-lung ventilation (OLV) of the right lung to avoid pressure loading on the left lung, and the tracheal tube was removed after confirming that air leakage had been managed. However, due to another massive air leak, we performed the same surgery again. As with the second postoperative period, the patient was managed for three days under OLV of the right lung; however, to ensure more stable management, we administered muscle relaxants in addition to analgesics and sedatives. After confirming that air leakage had been managed, the tracheal tube was removed, and there were no recurrences. In this case, the TachoSil^®^ patch was sutured in place to stabilize it at the site of air leakage until the pleural mesothelium grows on the surface of the TachoSil^®^ patch. This technique, in combination with management under OLV to reduce pressure loading on the affected lung, is expected to ensure the development of the pleural mesothelium. In this patient, the combination of the suturing TachoSil^®^ patch in place at the site of air leakage during surgery and temporary postoperative OLV was associated with the successful control of massive air leakage from vulnerable lung tissue.

## Introduction

Postoperative air leakage is one of the most common complications after pulmonary resection [1]. It occurs more frequently in patients with emphysematous lungs because of the vulnerability of the lung parenchyma and reduced tissue elasticity. However, there are no clear definitions or reports on the incidence of massive air leakage resulting in the uncontrollable progression of subcutaneous emphysema after lung resection. TachoSil^®^ (Corza Medical, Linz, Austria) is a sheetlike biological tissue adhesive and sealant consisting of an equine collagen patch coated with human fibrinogen and thrombin; it is used for hemostasis in cardiovascular surgery [2], the reconstruction of the ventricular wall in neurosurgery [3], and the control of air leakage in thoracic surgery. To control air leakages, a TachoSil^® ^patch is applied to the damaged tissue site, and the tissue repair occurs with mesothelial cell proliferation on the surface of the TachoSil^®^ patch [4]. However, the TachoSil^®^ patch may detach from the application site; in such cases, tissue repair may not progress, which could lead to a prolonged air leakage.

The "Tachosuture" technique (TT) has been reported as a technique for the prevention of prolonged air leakage, in which a TachoSil^®^ patch applied to the site of air leakage is sutured to prevent its displacement [5]. Although TT is effective for controlling prolonged air leakages, it is not a technique for controlling massive air leakages. For this reason, particularly when massive air leakages occur in vulnerable lung tissues, such as in cases of emphysema, even if TT is used to control the air leakage, the TachoSil^® ^patch may not stabilize the leak site, and the air leakage may not be controlled. We defined massive air leakage as an air leakage in which subcutaneous emphysema progresses rapidly despite adequate chest drainage, accompanied by continuous air leakage during both inspiratory and expiratory phases. We report a patient who developed massive air leakage after lobectomy and was managed using a combined strategy consisting of TT and postoperative one-lung ventilation (OLV). This approach minimized pressure loading to the affected lung, may have prevented the displacement of the TachoSil^®^ patch, and facilitated pleural healing. We believe that the combination of TT and OLV contributed to the successful control of the massive air leakage. This report presents the clinical course of this case and discusses the treatment strategy, its underlying rationale, and its limitations.

## Case presentation

The patient was a 50-year-old man with a smoking history of 76 pack-years. He had a history of drinking more than 1 L of beer per day and had alcoholic liver damage but no history of oral medication. He was diagnosed with an abnormal shadow on chest radiography during a health checkup, and a 29 mm-diameter nodule was detected in S6b of the left lung on chest computed tomography (CT) (Figure 1), which led to a visit to the department of respiratory medicine at our hospital. The carcinoembryonic antigen (CEA) and cytokeratin 19 fragment (CYFRA) levels were elevated as a tumor marker in the blood sampling test (Table 1), and a CT-guided biopsy confirmed the diagnosis of adenocarcinoma.

**Figure 1:**
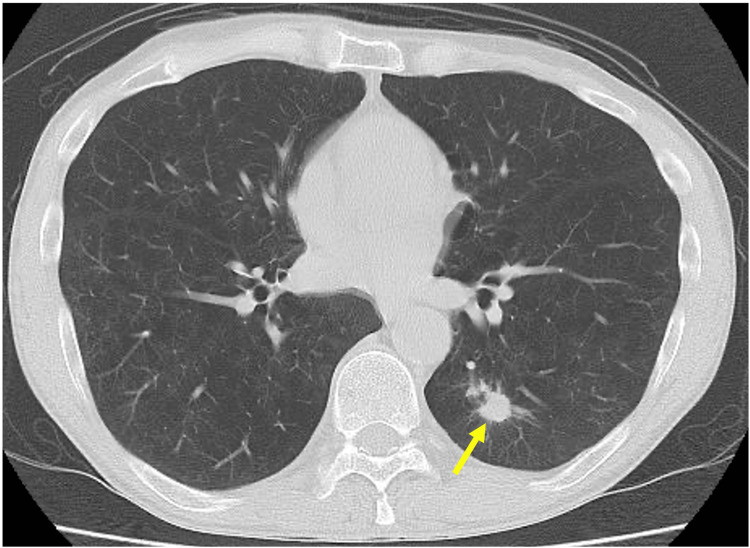
Preoperative chest computed tomography findings Chest computed tomography showed emphysema due to smoking and a 29 mm-diameter nodule in S6b of the left lung (arrow). The nodule was accompanied by spicules, but no pleural indentation was observed. Furthermore, no pleural effusion was observed

**Table 1:** Preoperative laboratory data WBC, white blood cells; RBC, red blood cells; Hb, hemoglobin; Plt, platelet; CRP, C-reactive protein; Alb, albumin; AST, aspartate transaminase; ALT, alanine transaminase; ALP, alkaline phosphatase; LDH, lactate dehydrogenase; γ-GTP, gamma-glutamyl transferase; AMY, amylase; BUN, blood urea nitrogen; Cre, creatinine; CEA, carcinoembryonic antigen; SLX, Sialyl-Lewis^X^ antigen; CYRFA, cytokeratin 19 fragment; ProGRP, progastrin-releasing peptide; PT, prothrombin time; INR, international normalized ratio

Variables	Patient values	Reference range
WBC	4.38	3.3-8.6 × 10^3^/μL
RBC	4.41	4.35-5.55 × 10^6^/μL
Hb	11.5	13.7-16.8 g/dL
Plt	12.2	15.8-34.8 × 10^4^/μL
CRP	0.12	<0.14 mg/dL
Alb	3.6	4.1-5.1 g/dL
AST	157	13-30 U/L
ALT	71	10-42 U/L
ALP	413	106-322 U/L
LDH	250	124-222 U/L
γ-GTP	273	13-64 U/L
AMY	155	44-132 U/L
BUN	10.7	8-20 mg/dL
Cre	0.61	0.65-1.07 mg/dL
Na	140	138-145 mmol/L
K	4.0	3.6-4.8 mmol/L
Cl	108	101-108 mmol/L
CEA	8.5	<5.0 ng/mL
SLX	33.1	<38.0 U/mL
CYFRA	4.6	<2.2 ng/mL
ProGRP	38	<81 pg/mL
PT%	53.4	80%-120%
PT-INR	1.51	0.88-1.12

^18^F-Fluorodeoxyglucose positron emission tomography (PET)/CT and contrast-enhanced magnetic resonance imaging of the head showed no obvious distant metastases, and the patient was referred to our department for surgery with a diagnosis of left lower lobe lung adenocarcinoma, cT1cN0M0 stage IA3 (eighth edition) [6]. The preoperative performance status was 0. However, pulmonary emphysema was observed on chest CT, and preoperative respiratory function tests showed a decreased forced expiratory volume percent in one second (FEV_1.0%_) to 51.62% and percent predicted diffuse capacity of the lung for carbon monoxide (%DL_CO_) to 34.9% (Table 2).

**Table 2:** Findings of the preoperative lung functional test and six-minute walk test VC, vital capacity; FVC, forced vital capacity; FEV_1.0_, forced expiratory volume in one second; DL_CO_, diffuse capacity of the lung for carbon monoxide; 6MWT, six-minute walk test

Variables	Patient values	Predicted values	Percent predicted values
VC	5.91 L	3.86 L	153.1%
FVC	5.87 L	3.86 L	152.1%
FEV_1.0_	3.03 L	3.38 L	89.6%
FEV_1.0%_	51.6%	71.7%	72.0%
DL_CO_	8.5 mL/minute/mmHg	24.2 mL/minute/mmHg	34.9%
6MWT	410 m	666 m	61.6%

For this reason, the possibility of the introduction of home oxygenation therapy or developing intractable air leakage postoperatively was anticipated. In addition, because the patient was found to have alcohol-induced liver damage (Table 1), there were concerns that surgical stress could lead to worsening liver function and delayed wound healing. Although respiratory function tests were inadequate, the patient's six-minute walk was 410 m (Borg score: 7-9) (Table 2), and his hepatic impairment was grade A according to the Child-Pugh classification; therefore, he was judged to be operatively tolerant, and surgery was performed.

The surgery involved a left lower lobectomy and mediastinal lymph node dissection using a four-port thoracoscopic approach (Figure 2a-1).

**Figure 2:**
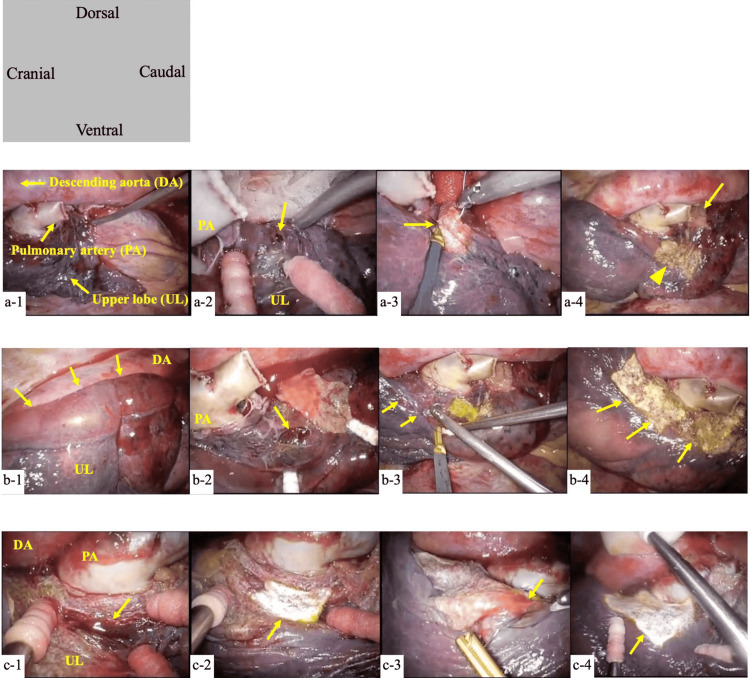
Findings of surgeries (a) First surgery: (a-1) The left lower lobe was resected, and the descending aorta and pulmonary artery stump were observed. (a-2) There was a defect in the visceral pleura in one area of the lung parenchyma of the interlobar plane (arrow), and air leakage was observed. (a-3) A TachoSil^®^ patch was applied over the pleural defect, and a single stitch was added over it (arrow). (a-4) In addition, a TachoSil^®^ patch was applied over it (arrow). An excess TachoSil^®^ patch was applied to reinforce the staple line of the lung parenchyma (triangular). (b) Second surgery: (b-1) A large cyst was observed on the interlobar plane (arrow). (b-2) A 2.0 cm-diameter tear in the cyst and air leakage from the tear were observed (arrow). (b-3) The TachoSil^®^ patch was applied over the pleural defect, two stitches were added over it, and the normal pleura at both ends of the cyst was sutured with seven stitches to sew the cyst inside (arrow). (b-4) In addition, a TachoSil^®^ patch was applied over the suture (arrow). (c) Third surgery: (c-1) A tear of the visceral pleura and air leakage from it were detected on the interlobar plane near the ventral side of the pulmonary artery (arrow). (c-2) A TachoSil^®^ patch was applied to the pleural defect (arrow). (c-3) The TachoSil^®^ patch was sutured with two stitches (arrow). (c-4) In addition, a TachoSil^®^ patch was applied (arrow)

In our department, air leakage in patients with severe emphysema is controlled by TT. After a left lower lobectomy, the air leakage site in the lung parenchyma on the interlobar surface (Figure 2a-2) was repaired using TT (Figure 2a-3, 2a-4), and the tracheal tube was removed in the operating room. No air leakage was observed in the operating room; however, immediately after admission to the intensive care unit (ICU), a cough-triggered massive air leakage was observed. Despite continuous suction at -20 cmH₂O, extensive subcutaneous emphysema rapidly progressed, spreading from the chest wall to the neck within minutes. Continuous air leakage was observed throughout both inspiratory and expiratory phases, suggesting a high-flow air leakage. The amount of air leakage was so large that subcutaneous emphysema could not be controlled by conservative treatment, and reoperation was performed on the same day.

The second surgery was also performed thoracoscopically, and a large cyst with a 2.0 cm-diameter pleural defect was found on the interlobar surface. Based on this finding, we concluded that the air leakage originated from a pneumatocele that developed on the interlobar surface after lobectomy (Figure 2b-1, 2b-2). Although we struggled with the method of controlling the air leakage because of his low pulmonary function, the decision was made to choose a surgical approach that avoided additional lung resection as far as possible. Therefore, the pleural defect was repaired with TT, the normal pleura at both ends of the cyst was sutured with seven stitches of absorbable thread to sew the pneumatocele inside (Figure 2b-3), and the TachoSil^®^ patch was further applied over the suture (Figure 2b-4). In addition, there was concern that postoperative pressure loading on the affected side could prevent the stabilization of the TachoSil^®^ patch and promote pneumatocele recurrence. For these reasons, the patient was admitted to the ICU without the removal of the tracheal tube, and OLV of the right lung was maintained under analgesia and sedation to avoid pressure loading on the left lung.

Buprenorphine was administered for analgesia at 5 μg/kg every eight hours, propofol was administered for sedation at 1.0 mg/kg/hour, and a 35 Fr double-lumen tube was used for OLV. The ventilator settings were set at an airway pressure of ≤30 cmH2O, positive end-expiratory pressure of 8-12 cmH2O, and pressure support of 5 cmH2O, and expectoration in the airway and repositioning were performed as appropriate. Antimicrobials were used only intraoperatively and not in the ICU. After admission to the ICU, the chest drain was managed on a water-seal system. No air leakage was detected immediately after ICU admission, presumably due to the ongoing OLV. After three days of OLV, the absence of an air leakage was confirmed under two-lung ventilation (TLV) with a chest tube connected to suction at -10 cmH₂O. Because of the prolonged sedation and OLV, careful weaning was performed over 24 hours, and the patient was extubated on postoperative day 4. Immediately after extubation, the patient experienced discomfort and hoarseness in the laryngeal region due to prolonged tracheal intubation, and vocal cord edema was observed. The immediate administration of methylprednisolone succinate sodium 125 mg resulted in good oxygenation without worsening vocal cord edema and no obvious neurological abnormalities. However, the day after the tracheal tube was removed, massive air leakage appeared again, triggered by coughing, and subcutaneous emphysema rapidly expanded. As emphysema was severe, and the serum albumin level was low, a third surgery was immediately performed, considering the possibility that the repair of the pneumatocele had failed.

The third surgery was also performed thoracoscopically and showed a tear and air leakage in the visceral pleura on a different interlobar plane (Figure 2c-1) but no pneumatocele. Based on these findings, we determined that even in this patient with vulnerable lung tissue and hypoalbuminemia, it was possible to control air leakage and prevent pneumatocele by repairing the lung tissue with TT and managing the patient with OLV. For this reason, after the repair of the visceral pleura with TT (Figure 2c-2, 2c-3, 2c-4), the patient was admitted to the ICU without the removal of the tracheal tube and was managed with OLV of the right lung (Figure 3a).

**Figure 3:**
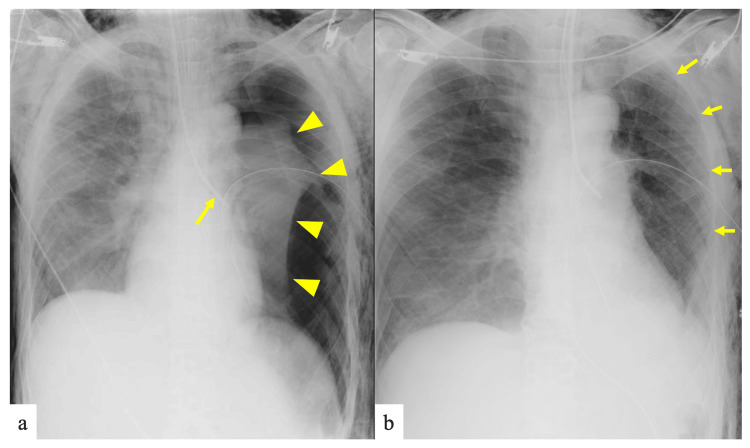
Chest X-ray findings (a) A chest radiograph taken immediately after the third surgery. A bronchial blocker is inserted in a single lumen tube (arrow), and the patient is on right one-lung ventilation (triangular). (b) This radiograph was taken after three days of one-lung ventilation, when the patient was on two-lung ventilation (arrow). After confirming that there was no air leakage, the endotracheal tube was removed the day after this radiograph was taken

At this time, OLV was performed using an 8.0 mm single-lumen tube with a smaller diameter than the 35 Fr double-lumen tube and a 9 Fr bronchial blocker to reduce the damage to the vocal cords. In addition to analgesics and sedatives, rocuronium has been used as a muscle relaxant to facilitate better management in the ICU. Antimicrobials were used only intraoperatively and not in the ICU. After the second surgery, the patient was managed with OLV for three days. Immediately after ICU admission, the chest drain was placed on water seal, and no air leakage was detected. After confirming the absence of an air leakage under TLV with a chest tube connected to suction at -10 cmH₂O, the tracheal tube was removed on postoperative day 4 (Figure 3b). No vocal cord edema was observed after extubation. Thereafter, no recurrence of air leakage was observed, and the patient was transferred to the general ward on the third day after extubation. The patient subsequently developed pneumonia on the affected side, which improved with antimicrobials, and there was no deterioration in the liver function. The patient was discharged on day 31 after the third surgery without the introduction of home oxygen therapy due to postoperative rehabilitation (Figure 4).

**Figure 4:**
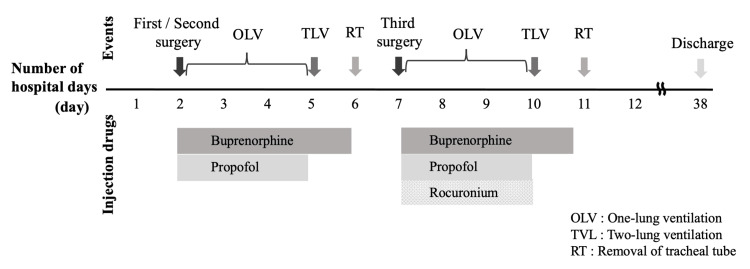
Clinical course

## Discussion

Although the incidence of air leakage after lung resection ranges from 28% to 60% and is not a rare complication, there are no reports on the incidence of massive air leakage with uncontrolled subcutaneous emphysema expansion [1]. If the expansion of subcutaneous emphysema can be controlled by thoracic drainage, conservative treatment for air leakage, such as pleurodesis or bronchial filling with the endobronchial Watanabe spigot, can be performed prior to surgery [7,8]. However, in cases of massive air leakage with uncontrolled subcutaneous emphysema expansion, conservative treatment is unlikely to be effective, and there is no time window for it. Therefore, prompt surgical treatment is considered; however, if the lung tissue is vulnerable, simple lung resection and suturing may have difficulty controlling air leakage, and a surgical method should be selected for each individual case [9,10]. In this case, although digital quantification was not available, the presence of continuous bubbling throughout the respiratory cycle, together with rapidly progressive subcutaneous emphysema despite -20 cmH₂O suction, indicated a high-flow air leakage. The emphysema extended from the chest wall to the cervical region within a short period, suggesting that the air leakage exceeded the capacity of the chest drainage system. Given the severity and rapid progression of these findings, conservative management was considered unlikely to achieve adequate control of the air leakage, and prompt reoperation was therefore indicated.

TachoSil^®^, a collagen matrix coated with lyophilized human fibrinogen and thrombin, is a surgical sealant that provides a mechanical scaffold for the healing process soon after surgery. TT is a technique to prevent prolonged air leakage, in which the surface of the TachoSil^®^ patch is sutured and fixed to prevent it from falling out until the regeneration of the pleural mesothelium occurs [4]. In the report by Nishida et al., postoperative OLV was not employed, and it is generally not required in typical cases [5]. However, in the present case, despite attempting to control the air leakage intraoperatively using TT, a massive air leakage developed postoperatively. This suggests that TT alone may have been insufficient to control the air leakage in this patient. In cases such as this, in which the lungs are vulnerable due to emphysema and hypoalbuminemia, a more stable strategy to maintain the fixation of the TachoSil^®^ patch may be required. Therefore, postoperative OLV was performed to reduce pressure on the affected lung and to minimize the risk of patch displacement. Although the mesothelium on the surface of the TachoSil^®^ patch can be confirmed histologically four days after application [4], all air leakage stopped within three days in the report by Nishida et al. [5], and we consider that three days was the shortest period of OLV that was acceptable.

Pneumatocele is a thin-walled cystic change within the lung and can occur after bacterial pneumonia in children, blunt chest trauma, or positive pressure ventilation, causing airway hemorrhage and infection [11]. Pneumatocele can range from asymptomatic to severe with infection and airway hemorrhaging but most often resolves spontaneously and is treated conservatively [12]. However, persistent air leakage and hemorrhaging require some form of treatment and additional surgical resection of the lung [13]. Because the present patient had low pulmonary function, it was necessary to avoid additional lung resection as much as possible. It has also been reported that the obstruction of the peripheral bronchi associated with the pressure compression of the lung parenchyma and the check valve mechanism are causes of pneumatocele, and we believe that our management strategy to avoid pressure loading on the affected side was effective [14].

This management strategy was conceptually based on the principle of reducing mechanical stress on suture sites through fasting and drainage management used in gastrointestinal surgery for postoperative suture failure. In the present case, postoperative OLV was performed to reduce mechanical stress on the affected lung. There is currently no direct evidence that the reduction of mechanical stress promotes the healing of the visceral pleura; thus, this remains a hypothetical mechanism. However, pleural repair is known to involve both the migration and proliferation of mesothelial cells [15]. Following pleural injury, mesothelial cells migrate across the denuded surface and subsequently proliferate to restore mesothelial continuity. The reduction of mechanical stress by OLV may have facilitated this reparative process by minimizing the repetitive disruption of the injured visceral pleura. OLV can be achieved either by using a double-lumen tube or by inserting a bronchial blocker into a single-lumen tube. When a double-lumen tube is used, expectoration in the airway is easy, and the lumen can be checked using bronchoscopy. However, its diameter is large, and the tube tip is implanted in the main bronchus; therefore, there is a risk of damage to the glottis and tracheal bifurcation [16]. Conversely, the insertion of a bronchial blocker in a single-lumen tube reduces these risks; however, it is difficult on the affected side to expectorate in the airway and to check the lumen by bronchoscopy (Figure 5).

**Figure 5:**
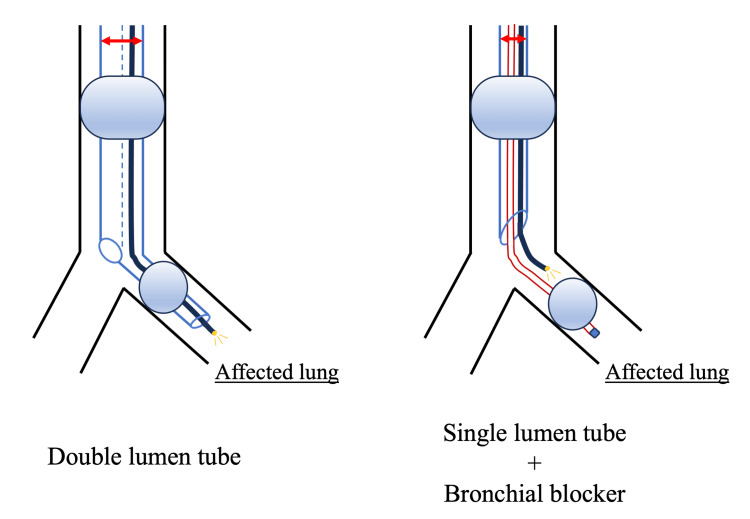
The difference between a double-lumen tube and a bronchial blocker inserted into a single-lumen tube Because a double-lumen tube has a larger diameter and the tube tip is placed in the main bronchus, there is a risk of damage to the glottis and the tracheal bifurcation. On the other hand, when a bronchial blocker is inserted into a single-lumen tube, it is difficult on the affected side to expectorate in the airway and to check the lumen by bronchoscopy. Furthermore, the larger outer diameter of double-lumen tubes may increase the risk of vocal cord injury. Image created using PowerPoint (Microsoft Corp., Redmond, WA)

The method of OLV should therefore be decided in consultation with the medical staff involved in the management, taking into account the individual characteristics of each case. The use of muscle relaxants, in addition to analgesics and sedatives, also reduced the risk of changing the position of the tracheal tube due to body movements, making the patient easier to manage in the ICU. Furthermore, the use of muscle relaxants in the present patient also enabled spontaneous breathing to be controlled, and pressure loading on the affected lung could be avoided more reliably; we therefore believe that this may be why air leakage did not recur after the third surgery. The pneumonia in this case was classified as hospital-acquired pneumonia and ventilator-associated pneumonia (VAP), as it developed more than 48 hours after hospital admission and the initiation of mechanical ventilation, respectively. Even in immunologically vulnerable high-risk patients, routine prophylactic antimicrobial administration for the prevention of VAP is not recommended [17]. Therefore, the use of prophylactic antimicrobials limited to the intraoperative period in this case was considered appropriate and consistent with current practice.

Although veno-venous extracorporeal membrane oxygenation can be used to rest the lungs, our management method is less invasive and has better medical economics [18]. In addition, the cost-effectiveness of TachoSil^®^ itself has been reported [19]. Our management strategy appears to be feasible in patients who can tolerate pulmonary resection and may represent a treatment option for massive air leakage. However, prolonged lung collapse for more than three days is considered a risk factor for re-expansion pulmonary edema, in addition to potential complications such as VAP, ICU-related syndrome, and muscle weakness associated with prolonged sedation and muscle relaxants [20]. In fact, the patient in this case developed VAP, which resolved with antimicrobial therapy. Because the patient was relatively young, the incidence of complications associated with prolonged mechanical ventilation and intensive care may have been limited. However, patients with vulnerable lung tissue are often older, and therefore, the indication for this strategy should be carefully evaluated on a case-by-case basis. Furthermore, management under OLV for a long period places a heavy burden on medical staff; therefore, adequate preparation, including the education of medical staff, is necessary.

## Conclusions

Massive air leakage following lung resection remains a critical challenge for thoracic surgeons. The combination of TT and OLV management may have more reliably prevented the detachment of the TachoSil^®^ path, potentially leading to the successful control of massive air leakage in patients with vulnerable lung tissue that is difficult to manage with TT alone. Although this strategy requires the careful consideration of the risks associated with prolonged mechanical ventilation and intensive care, it may represent a lifesaving option when conventional therapies fail. However, as this is a single-case report, definitive conclusions regarding its efficacy and safety cannot be drawn, and further studies are required.
